# Real-time imaging of hydrogen peroxide dynamics in vegetative and pathogenic hyphae of *Fusarium graminearum*

**DOI:** 10.1038/srep14980

**Published:** 2015-10-08

**Authors:** Michael Mentges, Jörg Bormann

**Affiliations:** 1 University of Hamburg, Biocenter Klein Flottbek, Department of Molecular Phytopathology and Genetics, Ohnhorststr. 18, D-22609 Hamburg, Germany

## Abstract

Balanced dynamics of reactive oxygen species in the phytopathogenic fungus *Fusarium graminearum* play key roles for development and infection. To monitor those dynamics, ratiometric analysis using the novel hydrogen peroxide (H_2_O_2_) sensitive fluorescent indicator protein HyPer-2 was established for the first time in phytopathogenic fungi. H_2_O_2_ changes the excitation spectrum of HyPer-2 with an excitation maximum at 405 nm for the reduced and 488 nm for the oxidized state, facilitating ratiometric readouts with maximum emission at 516 nm. HyPer-2 analyses were performed using a microtiter fluorometer and confocal laser scanning microscopy (CLSM). Addition of external H_2_O_2_ to mycelia caused a steep and transient increase in fluorescence excited at 488 nm. This can be reversed by the addition of the reducing agent dithiothreitol. HyPer-2 in *F. graminearum* is highly sensitive and specific to H_2_O_2_ even in tiny amounts. Hyperosmotic treatment elicited a transient internal H_2_O_2_ burst. Hence, HyPer-2 is suitable to monitor the intracellular redox balance. Using CLSM, developmental processes like nuclear division, tip growth, septation, and infection structure development were analyzed. The latter two processes imply marked accumulations of intracellular H_2_O_2_. Taken together, HyPer-2 is a valuable and reliable tool for the analysis of environmental conditions, cellular development, and pathogenicity.

*Fusarium graminearum* (teleomorph *Gibberella zeae*) is a necrotrophic, filamentous ascomycete. It infects all cereals and is one of the most important phytopathogens worldwide. Fusarium Head Blight (FHB) of wheat (*Triticum aestivum*) and barley (*Hordeum vulgare*) and ear rot of maize (*Zea mays*), caused by *F. graminearum*, are of special economic importance in terms of crop losses and accumulation of mycotoxins (reviewed in Sutton, 1982^1^; Goswami and Kistler, 2004^2^). *Fusarium graminearum* forms specialized infection structures, called infection cushions, in order to penetrate the surface of wheat floral leafs^3^. Their formation is important for colonization of the host as a *F. graminearum* adenylyl cyclase deletion mutant—defective in infection cushion development—fails to penetrate wheat epidermal cells^4^. Inside infection cushions, biosynthesis of trichothecenes takes place^3^. Penetration of the plant surface is accompanied by an unspecific plant response leading to necrosis directly underneath an infection cushion. Infection analysis using a trichothecene-deficient strain revealed that trichothecenes are neither necessary for penetration nor responsible for the formation of necrotic lesions in the plant^3^. Plant necrosis is often related to the production of reactive oxygen species (ROS)^5^. Reactive oxygen species are obvious by-products of aerobic life. They act in a harmful way on membranes, cell walls, proteins, nucleic acids and many other cellular components but, furthermore, also play a certain role in signal transduction. As a matter of first-line defense to invading pathogens, ROS are often produced and secreted by plants (reviewed in Glazebrook, 2005^6^; Heller and Tudzynski, 2011^7^). This immediate and unspecific response, called the oxidative burst, in turn, favors the infection of necrotrophic fungi, since they feed from dead plant material. *Fusarium graminearum* is widely recognized as a necrotrophic pathogen. However, controversy is ongoing whether or not there might be a brief biotrophic phase early in infection (reviewed in Kazan *et al.* 2012^8^). Given this ambiguity it is, to date, not unequivocally known whether or not *F. graminearum* faces an oxidative burst from the plant during penetration. Balanced production, secretion, and decomposition of ROS are part of the attack strategy of plant pathogenic fungi^5^,^7^,^9^. Interferences in the ROS-balance alter the pathogenic potential of *F. graminearum*. Disruption of FgOS-2, the HOG1-homologue of *F. graminearum*, leads to abnormal catalase gene expression and inappropriate accumulation of ROS. Ultimately, this causes malformed infection cushions (J. Bormann and W. Schäfer, unpublished results) and a drastic reduction in virulence on wheat^10^.

In order to elucidate the ROS-status in fungal mycelia, we established a novel fluorescence bioassay using the genetically encoded fluorescent indicator called HyPer^11^. Using bio-imaging techniques we analyze ROS-dynamics in mycelia of *F. graminearum in vitro* and in the early infection stages on wheat. HyPer consists of a circularly permuted yellow fluorescent protein (cpYFP) inserted into the regulatory domain (RD) of the prokaryotic H_2_O_2_-sensing protein, OxyR. Studies from HeLa cells prove a high specificity of this indictor for H_2_O_2_^11^,^12^ due to a hydrophobic pocket within OxyR that prevents the attack by charged oxidants such as the superoxide anion radical but allows the penetration of amphiphilic H_2_O_2_^13^. Upon oxidation, the formation of a disulfide bond mediates a conformational change inside OxyR-RD that is passed on to cpYFP. Oxidation of HyPer, thereby, increases fluorescence of cpYFP excited at 488 nm and decreases fluorescence excited at 405 nm, respectively. Maximal fluorescence emission is recorded at 516 nm. The sensory functions of HyPer are affected by the ambient pH. To circumvent false readouts and conclusions, a H_2_O_2_ insensitive variant of HyPer, called SypHer, was introduced^14^. A point mutation in one of the two H_2_O_2_-sensing cysteine residues of the OxyR-RD domain of Hyper renders the sensor unresponsive to H_2_O_2_, while preserving its pH sensitivity.

This is the first report on HyPer-fluorescence assays in a phytopathogenic fungus. Its expression in hyphae of *F. graminearum* provides insights in H_2_O_2_-dynamics inside mycelia of this destructive pathogen.

## Results

### HyPer-fluorescence responds specifically to varying amounts of external H_2_O_2_

**Figure 1:**
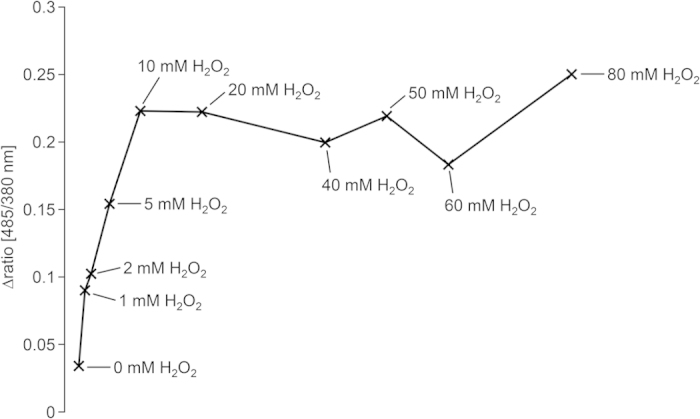
HyPer sensitivity assay. Assay for the relative changes in the ratio [485/380 nm] in response to increasing H_2_O_2_ concentrations. Mycelia were raised in a 96-well plate filled with 100 μl minimal agar medium and analyzed in a fluorometer. Prior to H_2_O_2_ injection 100 μl H_2_O were injected in each well.

*Fusarium graminearum* HyPer and SypHer mutants generated by protoplast transformation of the wild type PH1 were phenotypically characterized regarding vegetative growth, virulence, stress tolerance and fluorescence intensity. Three mutants with strong HyPer (herein referred to as PH1-HyPer mutants) and SypHer (herein referred to as PH1-SypHer mutants) fluorescence in the cytosol, respectively, were selected. Those mutants were similar to wild type regarding growth habit and sensitivity towards oxidative stress (Figure S1). To test responsiveness and specificity of HyPer-2, a microtiter plate assay using a fluorometer was established. An injector attached to the fluorometer facilitates injection of oxidizing and reducing agents, while, simultaneously, measuring fluorescence of mycelia grown on solid minimal medium (MM). A typical measurement cycle comprises the following steps: 1. measurement of ground-state fluorescence (in a range from 508 nm to 548 nm) after excitation at 380 nm and 485 nm, 2. fluorescence after injection of a test substance (e.g. H_2_O_2_), 3. fluorescence after injection of a second test substance (e.g. dithiothreitol, DTT), 4. if applicable, fluorescence after an additional injection (e.g. H_2_O_2_). Afterwards, the ratio of fluorescence recorded after excitation at 485 nm and 380 nm (herein after referred to as ratio [485/380 nm]) was calculated. Initially, we tested the sensitivity of HyPer-2 towards H_2_O_2_ in hyphae of *F. graminearum*. For this, increasing amounts of H_2_O_2_ (0, 1, 2, 5, 10, 20, 40, 60, 80 mM (here and from hereon, concentrations are given as final concentrations in the well)) and DTT (0, 5, 10, 20, 40, 50 mM) were added to fungal cultures in the microtiter plate. The ratio [485/380 nm] increased after injection of H_2_O_2_ at concentrations up to 10 mM (Fig. 1). Further increment of H_2_O_2_-concentrations did not evoke a higher ratio [485/380 nm] indicating a state of maximum oxidation in the presence of 10 mM H_2_O_2_. Viability of fungal hyphae was not compromised after treatment with up to 80 mM H_2_O_2_ (Figure S2). The reduction of HyPer by DTT increased proportionally to the amount of DTT added to the culture. The addition of 10 mM DTT moderately decreased the ratio [485/380 nm]. With higher DTT concentrations, the decrease in ratio [485/380 nm] got more pronounced (Figure S3).

**Figure 2:**
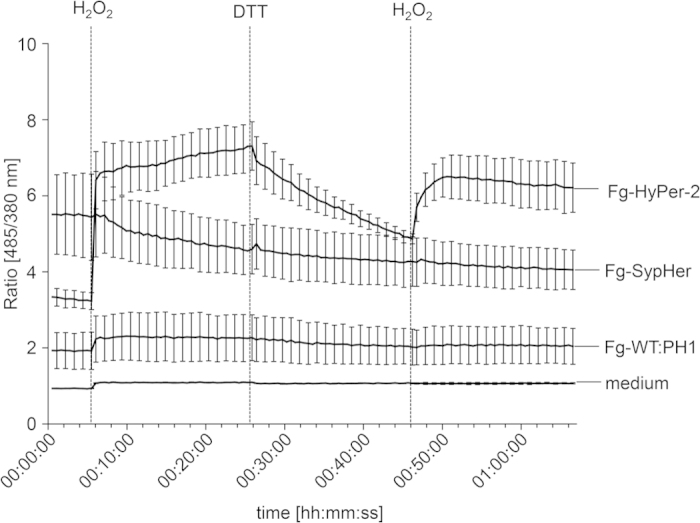
Ratiometric time course assay of the fungal response to external H_2_O_2_ and dithiothreitol (DTT). Timing of H_2_O_2_ (50 mM) and DTT (50 mM) induced ratio [485/380 nm] change in hyphae expressing HyPer-2, SypHer compared to the wild type and media control. Mycelia were raised in a 96-well plate filled with 100 μl minimal medium agar and analyzed in a fluorometer. Prior to the first H_2_O_2_ injection 100 μl H_2_O were injected in each well. Error bars represent the standard deviation (n = 24).

After sequential addition of 50 mM H_2_O_2_ and 50 mM DTT according to the scheme described above, no significant changes in ratio [485/380 nm] were observed in mycelia of the wild-type and PH1-SypHer and in the media control throughout the entire experimental procedure. This indicates that neither media nor wild type hyphae autofluorescence alters upon H_2_O_2_ or DTT injection and that SypHer, indeed, does not respond to oxidation and reduction. The PH1-HyPer mutants, in contrast, immediately responded to the injection of H_2_O_2_ with an up to 2-fold increase in the ratio [485/380 nm] from 3.2 (±0.22) to 6.4 (±0.75) (Fig. 2). As expected^11^, the fluorescence intensity after excitation at 380 nm (reduced state) moderately decreased, while the fluorescence emitted upon excitation at 485 nm (oxidized state) strongly increased (Figure S4). The ratio [485/380 nm] remained on a high level, presumably due to the fact that the H_2_O_2_ was not washed out from the wells. Addition of DTT decreased the ratio [485/380 nm] 1.5-fold compared to the maximum after oxidation. The second injection of H_2_O_2_ again increased the ratio [485/380 nm] on levels 1.2-times higher than the level before the first injection. Taken together, these results prove that HyPer-2 is responsive to oxidizing and reducing agents, and suitable for monitoring redox-fluctuations in *F. graminearum*.

### Environmental stimuli induce oxidative bursts in mycelia of HyPer-expressing hyphae

**Figure 3:**
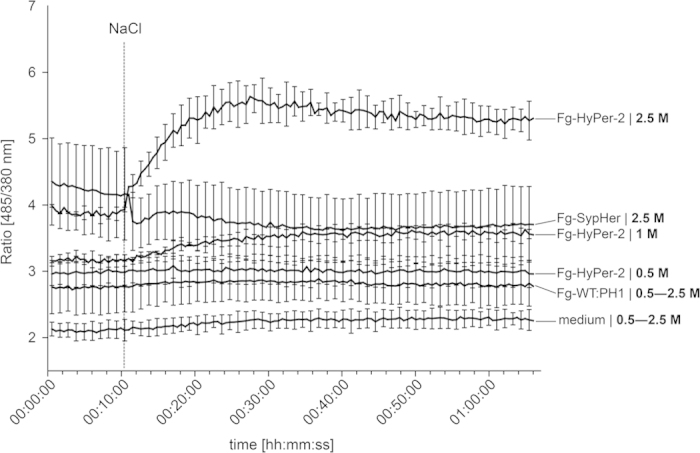
Ratiometric time course assay of the fungal response to osmotic stress. Timing of NaCl induced ratio [485/380 nm] change in hyphae expressing HyPer-2, SypHer compared to the wild type and media control. Mycelia were raised in a 96-well plate filled with 100 μl minimal agar medium and analyzed in a fluorometer. Prior to the NaCl injection 100 μl H_2_O were injected in each well. Error bars represent the standard deviation (n = 24). Values for the wild type and media control measurements after injection of NaCl are averaged.

To test the potential of HyPer-2 to report changes in the internal H_2_O_2_ level, NaCl^15^ was tested in the microtiter plate assay. The addition of NaCl increased the ratio [485/380 nm] of HyPer-2. This increase was proportional to the amount of NaCl added to the culture. While addition of 0.5 M NaCl did not change ratio [485/380 nm], it raised from 3.1 (±0.04) to 3.6 (±0.1) and from 3.9 (±0.04) to 5.5 (±0.33) after addition of 1 M and 2.5 M NaCl, respectively (Fig. 3). The increase in ratio [485/380 nm] in response to osmotic stress indicates a release of H_2_O_2_ inside the hyphae. This effect is H_2_O_2_-specific, since the addition of equimolar amounts of NaCl to PH1-SypHer did not alter the ratio [485/380 nm] (Fig. 3).

Taken together, these results show that HyPer is a highly specific sensor to monitor H_2_O_2_ release and subsequent decomposition upon external stresses in hyphae of *F. graminearum*.

### Life cell imaging for real-time monitoring of redox states in hyphae of *F. graminearum*

**Figure 4:**
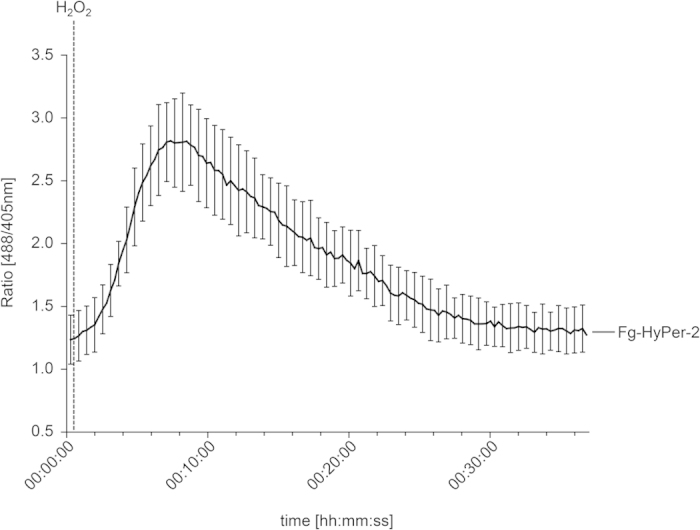
Ratiometric time course assay of the fungal response to external H_2_O_2_. Timing of H_2_O_2_ (50 mM) induced ratio [488/405 nm] change in hyphae expressing HyPer-2. Pixel intensities were measured in three regions of interest marked in a confocal laser scanning time series (see video S1). Error bars represent the standard deviation (n = 3).

To analyze HyPer-2-responses on the cellular level confocal laser scanning microscopy (CLSM) was performed. For this, a microfluidic chamber was developed that allows injection of stress agents to mycelia growing on agar, while, simultaneously, recording fluorescence (see methods section for details; Figure S5). After 24 h of growth in the flow chamber the conidia were germinated and the assay was started (n = 8). Injection of H_2_O_2_ caused an immediate increase in fluorescence after excitation at 488 nm, indicating a rapid oxidation of HyPer-2. Accordingly, fluorescence excited at 405 nm decreased (Figure S6). The ratio between both channels (ratio [488/405 nm]) increased from approximately 1 to a maximum of 2.8 (±0.35) followed by a slow decrease (Fig. 4, see also video S1). Hence, HyPer-2 enables real-time imaging of rapid oxidative processes inside fungal hyphae using fluorescence microscopy.

**Figure 5:**
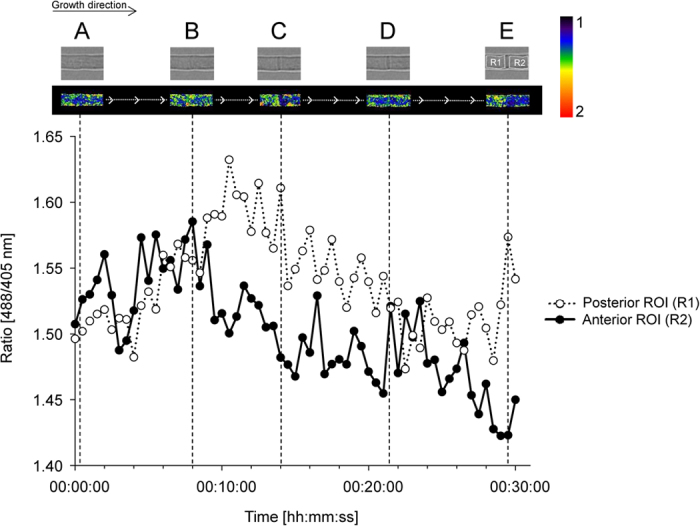
Ratiometric time course assay of septum formation in hypha expressing HyPer-2. Timing of H_2_O_2_ fluctuations during septum formation. Ratio [488/405 nm] calculated from pixel intensities measurements over time in two regions of interest (ROI R1, covering the posterior part and ROI R2, covering the anterior part of the hypha) marked in a confocal laser scanning time series (see also video S4). Annotations (**A–E**) depict certain developmental stages further explained in the text. Experiments were performed 6 times and gave similar results.

Using time-lapse microscopy we analyzed redox fluctuations during growth of fungal hyphae. These analyses included tip growth (n = 10), septum formation (n = 6), and nuclear divisions (n = 10). For the latter we took advantage of a PH1-HyPer-2 mutant expressing a copy of the histon-1 gene tagged with the fluorophore mCherry (A.L. Martínez-Rocha, unpublished results). This facilitates imaging of nuclear dynamics. Fungal growth was imaged over at least one hour taking pictures every 30 s (image acquisition for each channel took 10 s including filter exchange). *F. graminearum* hyphae were growing with a constant rate of around 1.25 μm min^−1^. No spatially distinct accumulations of H_2_O_2_ were detectable in the growing tip of *F. graminearum* (Figure S7 and video S2). Hence, establishment of polarity axes in hyphal growth seems not to involve H_2_O_2_. Also nuclear divisions during hyphal growth did not provoke fluctuations in intracellular H_2_O_2_ concentrations (Figure S8 and video S3). Septum formation, in contrast, went along with spatially distinct H_2_O_2_ accumulations (Fig. 5, Figure S9 and video S4). To measure fluorescence intensities, regions of interest of equal size were defined both to the right and the left of the anticipated location of the septum, representing the anterior and posterior half of the observation area. Prior to septum formation (range between point A and B in Fig. 5), the ratio [488/405 nm] was nearly similar throughout the observation area. After 9 min of observation (shortly after time point B in Fig. 5 and approximately 4 min after initiation of septum formation, indicated as dotted line in Figure S9), the ratio [488/405 nm] in the anterior part of the hyphae decreased and indicated lower amounts of H_2_O_2_ compared to posterior part that appeared to be more oxidized. 16 min after initiation of septum formation, the ratio [488/405 nm] is again balanced between both parts of the hyphae (time point D in Fig. 5). Interestingly, 23 min after initiation, the posterior part again got more oxidized than the anterior part (time point E in Fig. 5). Similar experiments using PH1-SypHer did not reveal any significant differences between the ratios [488/405 nm] in the anterior and posterior part of the hyphae (Figure S10).

These results demonstrate that real-time imaging using HyPer represents a valuable tool to gain a deeper understanding of cell biology in fungi.

### HyPer fluorescence assay reveals higher H_2_O_2_-levels in infection cushions of *Fusarium graminearum*

**Figure 6:**
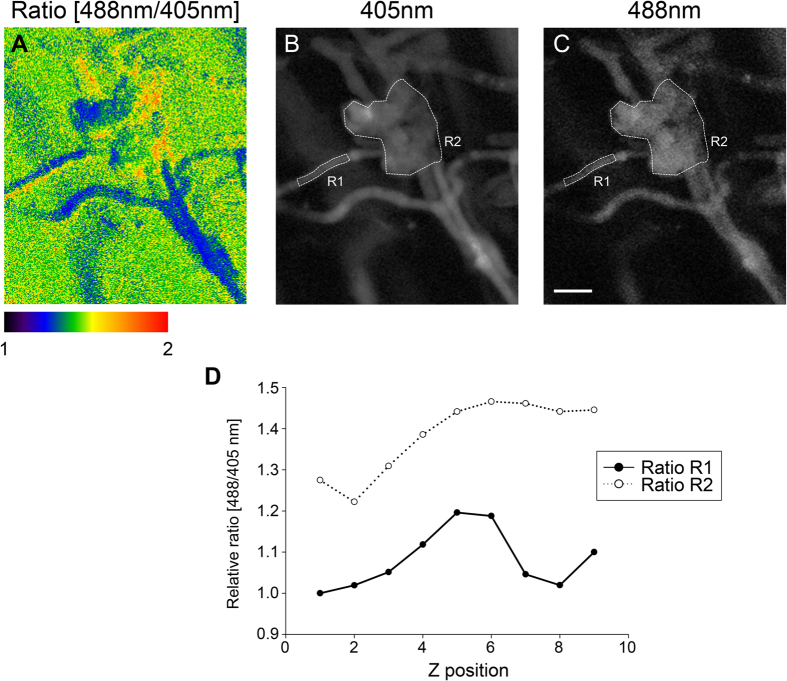
Ratiometric assay of infection cushion formation in hypha expressing HyPer-2. (**A–C**) Average intensity projections of a tiny infection cushion developed on a wheat palea 6 days post inoculation. **(A**) Ratio [488/405 nm]. (**B**) Fluorescence excited at 405 nm. (**C**) Fluorescence excited at 488 nm. (**D**) Pixel intensities were measured over nine z-positions in two regions of interest (ROI R1, covering a part of a runner hypha and ROI R2, covering a part of an infection cushion) marked in the confocal laser scanning z-series shown in (**A,B**). Analysis of ten different infection structures gave similar results. Scale bar 10 μm.

Infection cushion development can be induced on detached wheat floral organs^3^. Using fluorescent reporter strains, biogenesis of infection cushions emerging from non-infective runner hyphae can be followed over time using CLSM. As expected, the PH1-HyPer mutants developed infection cushions on palea in a wild-type like manner starting 4-5 days post inoculation (dpi). Ratiometric HyPer imaging revealed a greater ratio [488/405 nm] in infection cushions indicating relatively higher H_2_O_2_-concetrations compared to runner hyphae (Fig. 6A). Figure 6 serves as an example of ratiometric analysis of infection structures and is representative for a series of similar results. For ratio [488/405 nm] calculation regions of interest were defined covering parts of the runner hyphae and the infection cushion (Fig. 6B and C). The ratio [488/405 nm] was approximately 1.3 to 1.5 times the ratio [488/405 nm] of the runner hyphae (Fig. 6D). Therefore, we conclude that the fungus faces localized H_2_O_2_ accumulations specifically in plant-penetrating infection structures. Similar experiments using the PH1-SypHer strains showed no marked differences in the ratio [488/405 nm] between the runner hyphae and the infection cushions and within either structure (Figure S11).

In conclusion, ratiometric analyses of H_2_O_2_-fluctuations using HyPer-2 offers new insights to regulatory networks and complex interplays between pathogens and their hosts. Furthermore, they are valuable complements to classical genetic approaches for the analysis of cell biology in fungi.

## Discussion

In the present study we analyzed oxidative processes in vegetative and infective hyphae of *F. graminearum*. To achieve this, we, for the first time in a phytopathogenic fungus, used the ratiometric fluorescent H_2_O_2_-indicator HyPer-2 to monitor fluctuations in H_2_O_2_-level inside hyphae.

HyPer-2 enables monitoring of fluctuations in the intracellular H_2_O_2_-level in response to external stimuli, genetic modifications, and during different developmental stages like nuclear division and septum formation. Ratiometric HyPer-2 imaging uses a genetically encoded sensor protein and, therefore, is equally distributed in the cell or can be targeted to a certain subcellular compartment, e.g. the mitochondria^16^. This makes it advantageous over classical staining methods like 2′,7′-dichlorodihydrofluorescein diacetate (H_2_DCFDA), 3,3′-Diaminobenzidine (DAB), or boronate-based H_2_O_2_ probes (reviewed in Guo *et al.* 2014^17^). Technically sophisticated application, insufficient uptake, and inadequate intracellular distribution of stains can, therefore, be avoided. Boronate-based probes are fluorescence turn-on probes with high specificity to H_2_O_2_. They have been widely used for live-cell imaging in non-cell wall systems^18^. Ledoux *et al.* (2013)^19^ describe the application of this probe in *Arabidospsis thaliana*. However, enhancement of fluorescence upon oxidation of boronate-based dyes is irreversible and, therefore, limits its applicability for analysis of H_2_O_2_ dynamics. Many other stains (e.g. DAB) do not allow live cell imaging since their application requires harsh conditions like ethanol fixation.

In addition, HyPer-2 has some advantages over other genetically encoded fluorescent indicators. The redox-sensitive GFP2, sometimes coupled to glutaredoxin (GRX), is widely used to analyze redox states in cells indirectly by measuring the thiol redox potential triggered by the electron flow between glutathione and roGFP2^20^,^21^. Glutathione is an important and ubiquitous antioxidant. It contains a thiol group that reversibly gets oxidized (forming glutathione disulfide) or reduced by the action of GRX. As depicted before, these indicators do not immediately monitor the redox status of a cell or fluctuation in the H_2_O_2_-concentrations. This limitation can be overcome by in-frame fusion of roGFP2 to the Orp1 peroxidase with high specificity to H_2_O_2_^21^. However, it cannot be ruled out that constitutive overexpression of GRX or Orp1 coupled to roGFP2 alters the cellular redox status, since both proteins display enzymatic activity in the cell. In HyPer-2, the prokaryotic H_2_O_2_-sensing domain OxyR mediates the conformational change within the cpYFP moiety leading to ratiometric readouts. There are no homologues of this domain in the genome of *F. graminearum* (e-value: <1; data not shown). Hence, HyPer-2 does not interfere with the intracellular ROS-balance and its readout does not rely on enzymatic reactions. Given its biophysical properties it is assumed that HyPer-2 itself, theoretically, can act as an antioxidant^13^. However, studies from mammalian systems prove that even strong expression does not interfere with the intracellular ROS-level^22^,^23^. Previous analyses indicate that the ambient pH is an issue for HyPer-2 measurements (reviewed in Meyer and Dick, 2010^24^). To rule out any pH-effect in our measurement, we constitutively expressed a H_2_O_2_-insentive HyPer-variant (called SypHer)^14^ in mycelia of *F. graminearum* and conducted several control experiments. In all assays the SypHer ratio did not significantly change indicating that the intracellular pH does not interfere with the measurements. Studies from *Neurospora crassa* demonstrate that the intracellular pH is tightly controlled and rapidly readjusted after perturbation, e.g. by application of acidic substances^25^.

Reactive oxygen species are unavoidable by-products of a plethora of biochemical processes in the presence of oxygen. Their production and decomposition is usually well balanced and interference with this balance often causes physiological disorders and disease (reviewed i.e. in Taverne *et al.* 2013^26^; Poljsak *et al.* 2013^27^). This insinuates a Janus-faced nature of ROS in cellular processes: opposing a deleterious impact of ROS on lipids, nucleic acids, and proteins they possess profitable function in cell signaling cascades. Localized production of ROS has recently been implicated in the determination of polarity axes and, therefore, in the regulation of directed growth^28^,^29^. Our results demonstrate the absence of a tip-high H_2_O_2_ accumulation in growing hyphae of *F. graminearum* (Figure S7 and video S2). This suggests that rather superoxide anions (O_2_^−^) accumulate in the hyphal tips. Supportive for this notion is that disruption of O_2_^−^ generation by functional inactivation of nicotinamide adenine dinucleotide phosphate (NADPH) oxidase (NOX) complex enzymes often cause abnormal apical growth and faulty determinations of polarity axis in fungi^30^.

In the field of phytopathology well balanced ROS-equilibria are also mandatory for proper host-pathogen interaction. Development and pathogenicity of *F. graminearum* relies on appropriate equilibration of ROS. The deletion of the stress-activated mitogen-acivated protein kinase *FgOS-2* and its downstream transcription factor *Fgatf1* interfere with ROS-decomposition processes. In consequence, these mutants are defective in maintaining the ROS equilibrium and are, therefore, strongly reduced in virulence^10^,^31^. More aggressive pathogens like *Botrytis cinerea* and *Leptosphaeria maculans*, in contrast, benefit from oxidative bursts derived from themselves and the host^32^,^33^. Given this diversity in regard to the importance of ROS in host pathogen interaction, we choose to investigate the actual redox state inside pathogenic hyphae.

It is described that *F. graminearum* preferentially enters wheat floral tissues through natural openings of the plant like stomata^34^,^35^. More recent findings, however, contradict this notion and substantiate the assumption that specialized and morphological distinct infection structures facilitate immediate penetration of the plant surface and that infection through stomata is more likely to occur stochastically^3^. These structures are structurally dissimilar from dome-shaped appressoria i.e. of *Magnaporthe oryzae* in which high turgor pressure is present^36^. *F. graminearum* infection structures appear as lobate, branched and frequently septated hyphal mats that, unlike *M. oryzae* appressoria, develop from epiphytically growing runner hyphae several days post inoculation and not from germinating conidia (see also Emmett and Parbery, 1975^37^; Mendgen *et al.* 1996^8^). For ratiometric analysis of redox states in infective hyphae, we took advantage of the clear morphological distinction between runner hyphae (non-infective) and infection cushions. As depicted in Fig. 6, HyPer is more oxidized in the infection cushion, indicating a local accumulation of H_2_O_2_. By use of roGFP-imaging, Heller *et al.* (2012)^38^ studied appressoria-like structures of *B. cinerea*. The authors also found a distinct oxidation inside these structures. Hence, localized oxidation inside fungal infection structures is a recurrent pattern in different phytopathogenic fungi that might, therefore, facilitate plant infection. This similar H_2_O_2_ accumulation in the infection structures of the necrotroph *B. cinerea* and *F. graminearum*, furthermore, indicate that also *F. graminearum* acts as a necrotoph already in the earliest infection stages. In the biotrophic fungus *Claviceps purpurea* which does not differentiate any specialized infection structures^39^, deletion of the O_2_^−^-generating NOX subunit NoxA leads to a drastic reduction in virulence. However, hyphae are still able to penetrate the plant^40^. In *M. oryzae*, in contrast, both NoxA and NoxB are mandatory for plant surface penetration^28^.

Recent results from *M. oryzae* indicate a scaffolding function of NOX-complex proteins in order to align septins and F-actin along the point of penetration peg emergence^41^. This involves localized ROS-production in cell-biological differentiation as it was proposed for developmental processes including sexual reproduction, polarized growth and infection structure development^28^ (reviewed in Scott and Eaton, 2008^42^). Yet, localized ROS-accumulation inside infection cushions of *F. graminearum* might promote their formation.

Considering that, at least in *M. oryzae*, synthesis of ROS by the NOX-complexes directly controls F-actin dynamics, it is reasonable to expect local fluctuations in H_2_O_2_ at the site of septum formation, a process that also implies localized recruitment of F-actin^43^. The fact that we also detected spatially distinct H_2_O_2_ accumulations indicates a similar functional connection between ROS-production and F-actin recruitment for septum formation in *F. graminearum*. However, thus far not much is known about the genetic and biochemical trigger of septum formation and its suppression. Advancements in recent research (reviewed in Harris, 2012^44^) have shown that localized ROS accumulations are involved in the regulation of septum formation but, undoubtedly, this phenomenon requires further investigation.

NBT staining revealed that growth under high salinity stress conditions increases ROS production^31^. Furthermore, mutants lacking the central regulator of oxidative stress response, the b-zip transcription factor AP1, display a relatively higher sensitivity towards salt stress^15^. By use of HyPer-imaging we now demonstrate the intracellular production of H_2_O_2_ in the presence of high amounts of salt in the medium (Fig. 3). This is in accordance to the transcriptional upregulation of potential catalase genes in response to salt stress^10^.

Reactive oxygen species are involved in a plethora of developmental processes in fungi and beyond. Using the fluorescent H_2_O_2_ reporter HyPer we, for the first time in a phytopathogenic fungus, introduce a highly specific and reliable tool to analyze H_2_O_2_ fluctuations in living cells. Application of HyPer imaging will assist analysis of stress responses and developmental processes in fungi.

## Methods

### Generation of plasmids

Standard recombinant DNA methods were performed according to Ausubel *et al.* (2002)^45^ and Green and Sambrook (2012) *et al.* (1989)^46^. For stable expression in mycelia of *F. graminearum*, the entire open reading frame (ORF) of HyPer-2 and SypHer was amplified by PCR using the Q5 proof reading polymerase (New England Biolabs, Frankfurt a. M., Germany) from plasmid pC1-Hyper-2^12^, using primers A1 and A2 (Table S1) and cloned in frame with the promoter of the glyceraldehyd-3-phosphate dehydrogenase from *Aspergillus nidulans* (*gpdA*-promoter) using *Bam*HI restriction sites introduced to the primers. Both plasmids (named p7-HyPer-2 and p7-SypHer) were used to transform the *F. graminearum* wild type PH1.

### Generation of mutants

Protoplast transformation of *F. graminearum* was performed as described previously^31^. Briefly, mycelia were grown overnight in 100 ml YEPG (0.3% yeast extract, 1% bacto peptone, 2% D-glucose), harvested by filtering, resuspended in 20 ml of enzyme solution containing driselase and lysing enzymes (Life Technologies, Darmstadt, Germany; 2.5%:0.5% in 0.6 M KCl) and incubated for 2–3 h at 30 °C. Undigested hyphal material was removed from the protoplast suspension by filtration. The protoplasts were pelleted by centrifugation at 6700 × g, washed once with 10 ml STC (20% sucrose, 10 mM Tris-HCl, pH 8.0, 50 mM CaCl_2_), centrifuged again, then resuspended and adjusted in STC at 1–6 × 10^8^ protoplasts per ml. For transformation, 200 ml of the protoplast suspension was mixed with approximately 6 μg of DNA and incubated at room temperature for 20 min. Subsequently, 1 ml PEG (40% polyethylene glycol 4000, 60% STC) was added and again incubated at room temperature for 20 min. The protoplast suspension was added to 50 ml TB3 agar (100 g sucrose, 0.3% yeast extract, 0.3% casamino acids, 1.5% agar) and poured into five 92 mm petri dishes. After 24 h, an overlay of TB3 agar (1.5%) containing 200 μg ml^−1^ hygromycin B was added to the plates. Putative transformants were obtained after 2 dpi at 28 °C. They were transferred to fresh plates of complete medium (CM; 1 l CM contained 10 ml of solution A (100 g l^−1^ Ca(NO_3_)_2_ × 4 H_2_O); 10 ml of solution B (20 g l^−1^ KH_2_PO_4_; 25 g l^−1^ MgSO_4_ × 7H_2_O; 10 g l^−1^ NaCl, sterilized by filtration); 10 g glucose; 1 ml of suspension D (60 g l^−1^ H_3_BO_3_; 390 mg l^−1^ CuSO_4_ × 5H_2_O;13 mg l^−1^ KI; 60 mg l^−1^ MnSO_4_ × H_2_O; 51 mg l^−1^ (NH_4_)_6_Mo_7_O_24_ × 4H_2_O; 5.48 g l^−1^ ZnSO_4_ × 7H_2_O; 932 mg l^−1^ FeCl_3_ × 6 H_2_O), 1 g yeast extract; 0.5 g enzymatically hydrolysed casein; 0.5 g acid-hydrolysed casein) supplemented with 250 μg ml^−1^ hygromycin B. Putative transformants with random integration of the construct were evaluated by fluorescence microscopy (data not shown). Those mutants with high expression of HyPer-2 and SypHer were used for further experiments.

### Fluorescence assays

Ratiometric analysis of vegetative hyphae was conducted in two ways: 1. Fluorometric measurements using multi-well plates and a fluorometer equipped with an injector (Biotek Synergy HT, Biotek, Bad Friedrichshall, Germany), and 2. confocal laser scanning microscopy (Zeiss Axio Imager Z2 with LSM 780 module, Zeiss, Oberkochen, Germany) using objective slides covered with CM-agar and equipped with a custom made injection system (Figure S5).

For fluorometer analysis, each well of black 96-well plates was filled with 100 μl MM-agar (1.5%) (MM; like CM, but without yeast extract and casein). After hardening, media were inoculated with 200 conidia of the wild type and mutant strains expressing HyPer-2 and SypHer, respectively. Fluorescence was exited at 385/10 nm and 485/10 nm and fluorescence emission was monitored at a range from 508 nm to 548 nm. In a typical experiment, fluorescence was recorded for approximately 5 min prior to injection of any agent in a given concentration. Each measure cycle after injection was approximately 20 min. Concentrations depicted in the Figure legends and the main text are given as final concentrations after dilution with medium or water present in the wells.

For fluorescence microscopy of *in vitro* mycelia, conidia of PH1-HyPer mutants were inoculated on objective slides covered with a thin layer of agar surrounded by a double-sided adhesive frame (Gene Frame, Thermo Scientific, Schwerte, Germany, see Figure S5). 12–14 hpi, a second frame was installed on top of the first that contained two openings which served as ports for injection and efflux of agents. A cover slip (24 × 40 mm) was placed on top of the second frame. Injection of agents was achieved by a syringe attached to a Heidelberger extension (Fresenius Kabi AG, Bad Homburg, Germany) and an endoneedle for root canal rinsing (Vedefar N.V., Dilbeek, Belgium).

For HyPer and SypHer imaging on inoculated wheat paleas, wheat florets were dissected using a razor blade, placed on water agar (1.5%) and inoculated with 100 conidia. Starting 7 dpi, paleas were screened for infection cushion development using a MZFLIII fluorescence stereomicroscope (Leica Microsystems, Heerbrugg, Switzerland). Positive samples were transferred to objective slides equipped with a Gene Frame and analyzed by CLSM. Regions of interest (ROIs) were defined inside runner hyphae and infection cushions and pixel intensities were measured.

HyPer and SypHer were both excited at 405 nm using a solid-state laser and at 488 nm using an argon ion laser. Fluorescence was recorded at a range from 508 nm to 548 nm. For calculation of ratios, regions of interest (ROIs) were defined in which pixel were counted. For ratiometric analysis, photo-multiplier sensitivity was adjusted in a way that excitation at 405 nm and 488 nm led to similar fluorescence intensities in the non-stressed situation, leading to a ratio of approximately 1.

## Additional Information

**How to cite this article**: Mentges, M. and Bormann, J. Real-time imaging of hydrogen peroxide dynamics in vegetative and pathogenic hyphae of *Fusarium graminearum*. *Sci. Rep.* **5**, 14980; doi: 10.1038/srep14980 (2015).

## Supplementary Material

Supplementary Figures

Supplementary Video S1

Supplementary Video S2

Supplementary Video S3

Supplementary Video S4
