# Supplementary material for: Real-time imaging of hydrogen peroxide dynamics in vegetative and pathogenic hyphae of Fusarium graminearum
Source: Sci Rep. 2015 Oct 8;5:14980. doi: 10.1038/srep14980 (PMC4597226; doi:10.1038/srep14980)
Supplement: Supplementary Figures [file srep14980-s1.pdf]

**Supplementary information**

# Real-time imaging of hydrogen peroxide dynamics in vegetative and pathogenic hyphae of *Fusarium graminearum*

Michael Mentges and Jörg Bormann

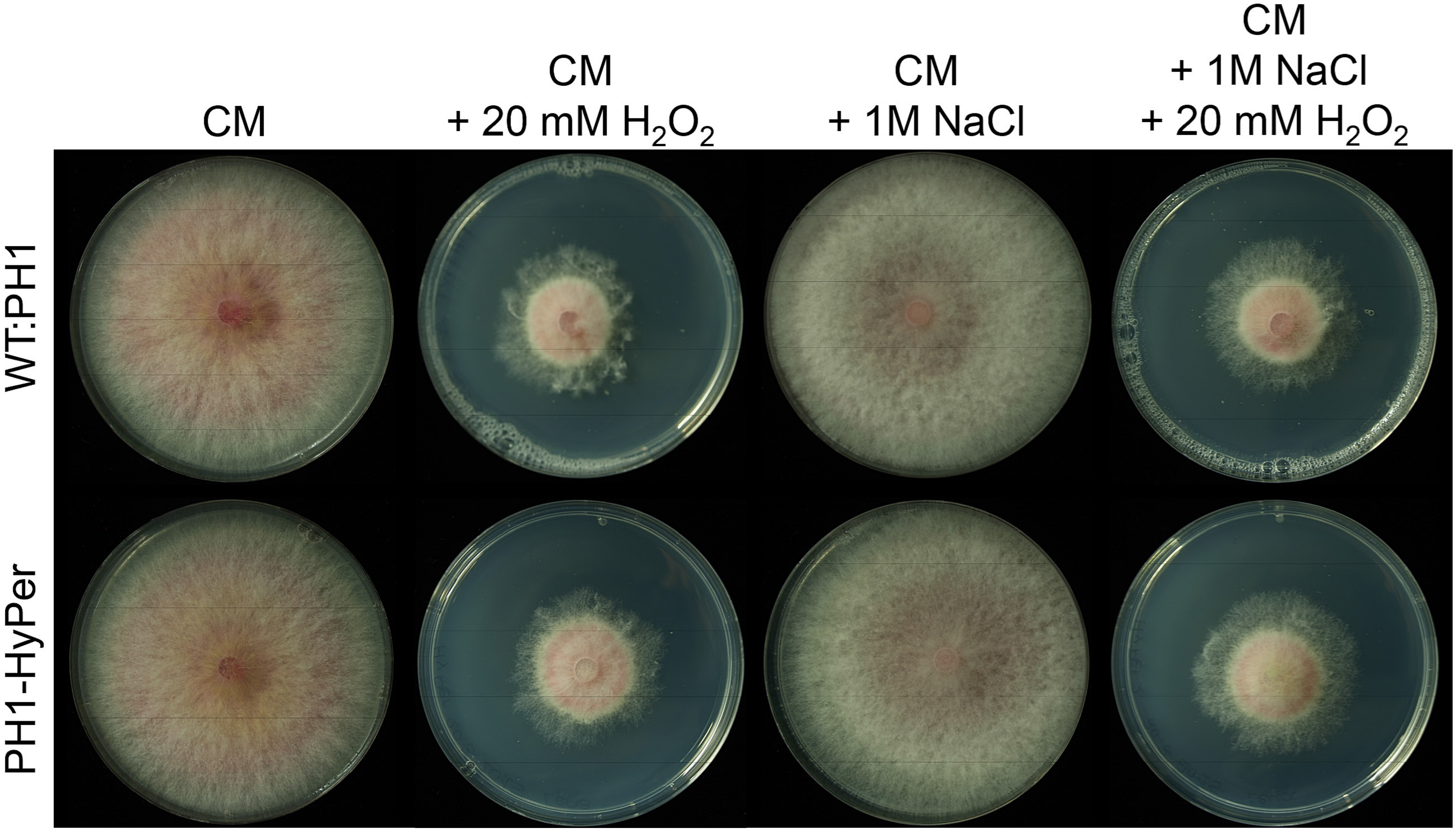


**Figure S1.** Colony morphology of wild type (WT:PH1) and HyPer-2-expressing strain (PH1-HyPer). Plate assay (4 dpi) of the wild type and PH1-HyPer on CM and CM supplemented with 20 mM $H_2O_2$ and 1 M NaCl, respectively, and both chemicals in combination.

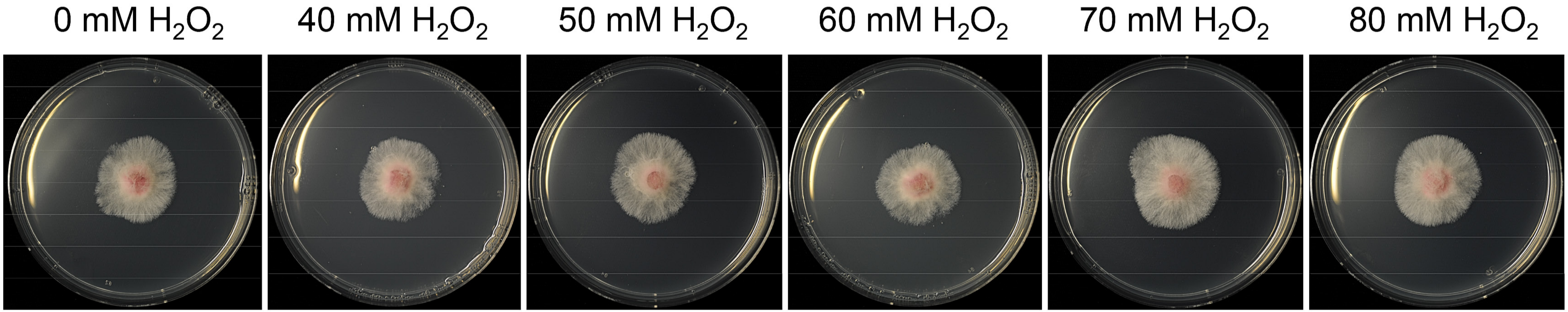


**Figure S2. Fungal viability assay.** Colony morphology (2 dpi) of the *F. graminearum* wild type on CM after treatment with increasing concentrations of $H_2O_2$. Inoculum was taken from microtiter plate assay after injection of $H_2O_2$ and fluorescence measurement for 45 min. Treatment with up to 80 mM $H_2O_2$ did not compromise fungal viability.

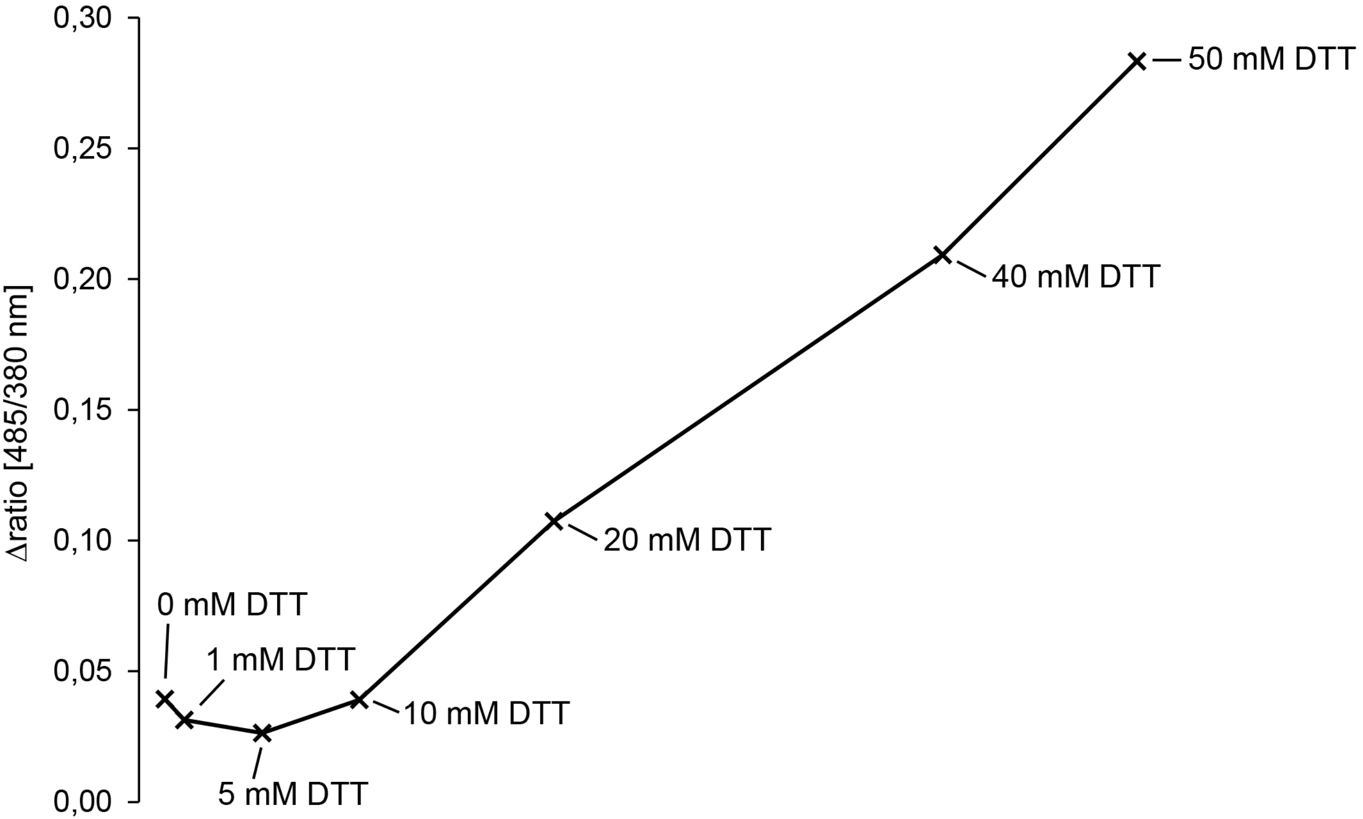


**Figure S3. HyPer sensitivity assay.** Assay for the relative changes in the ratio [485/380nm] in response to increasing dithiothreitol (DTT) concentrations. Mycelia were raised in a 96-well plate filled with 100 µl minimal agar medium and analyzed in a fluorometer. Prior to DTT injection 50 mM $H_2O_2$ were injected in each well.

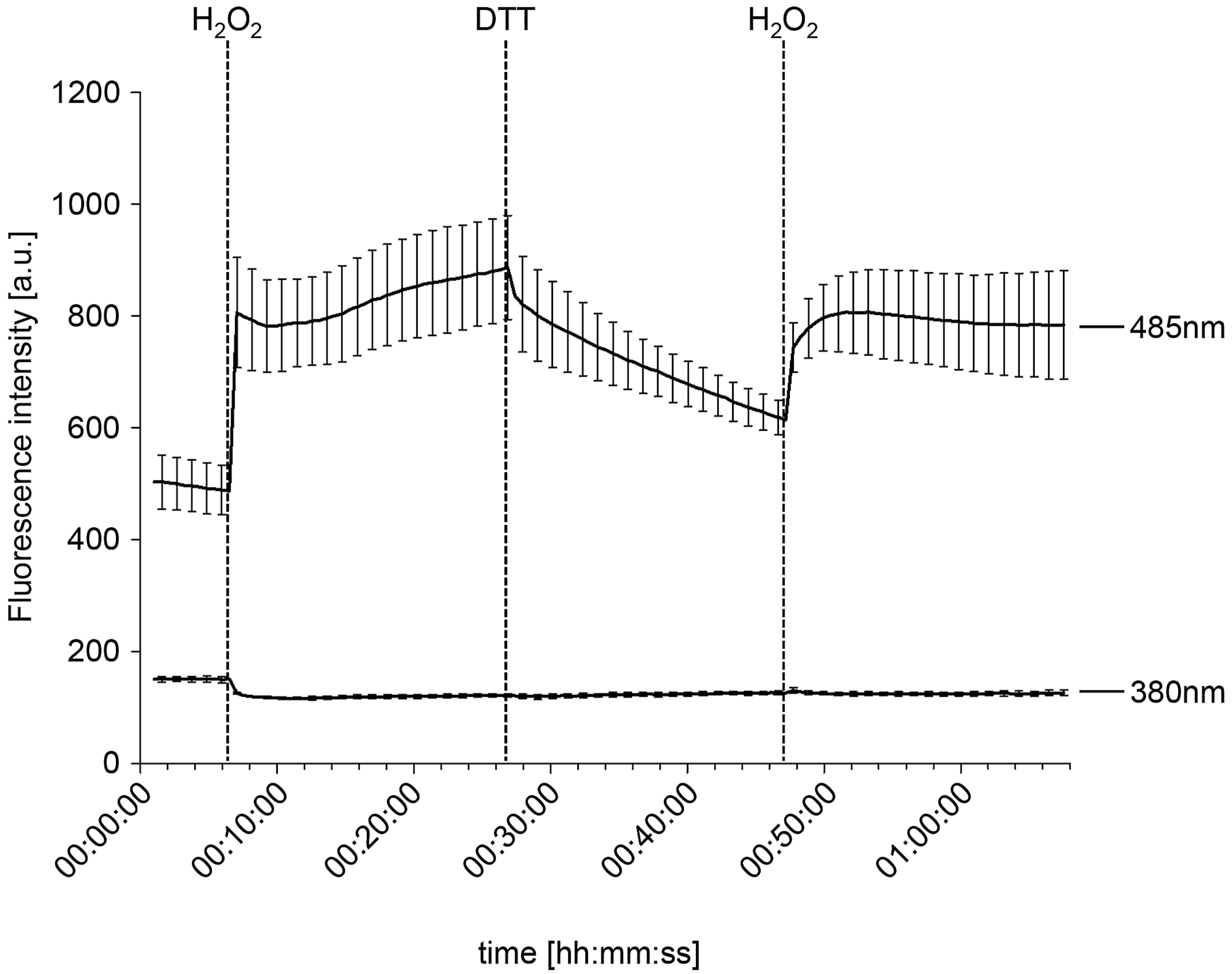


**Figure S4. Fluorescence time course assay of the fungal response to external $H_2O_2$ and dithiothreitol (DTT).** Timing of $H_2O_2$ (50 mM) and DTT (50 mM) induced changes in HyPer-2-fluorescence intensity after excitation at 380 nm and 485 nm, respectively. Mycelia were raised in a 96-well plate filled with 100 µl minimal agar medium and analyzed in a fluorometer. Prior to the first $H_2O_2$ injection 100 µl $H_2O$ were injected in each well. Error bars represent the standard deviation (n=24).

# Flow chamber preparation

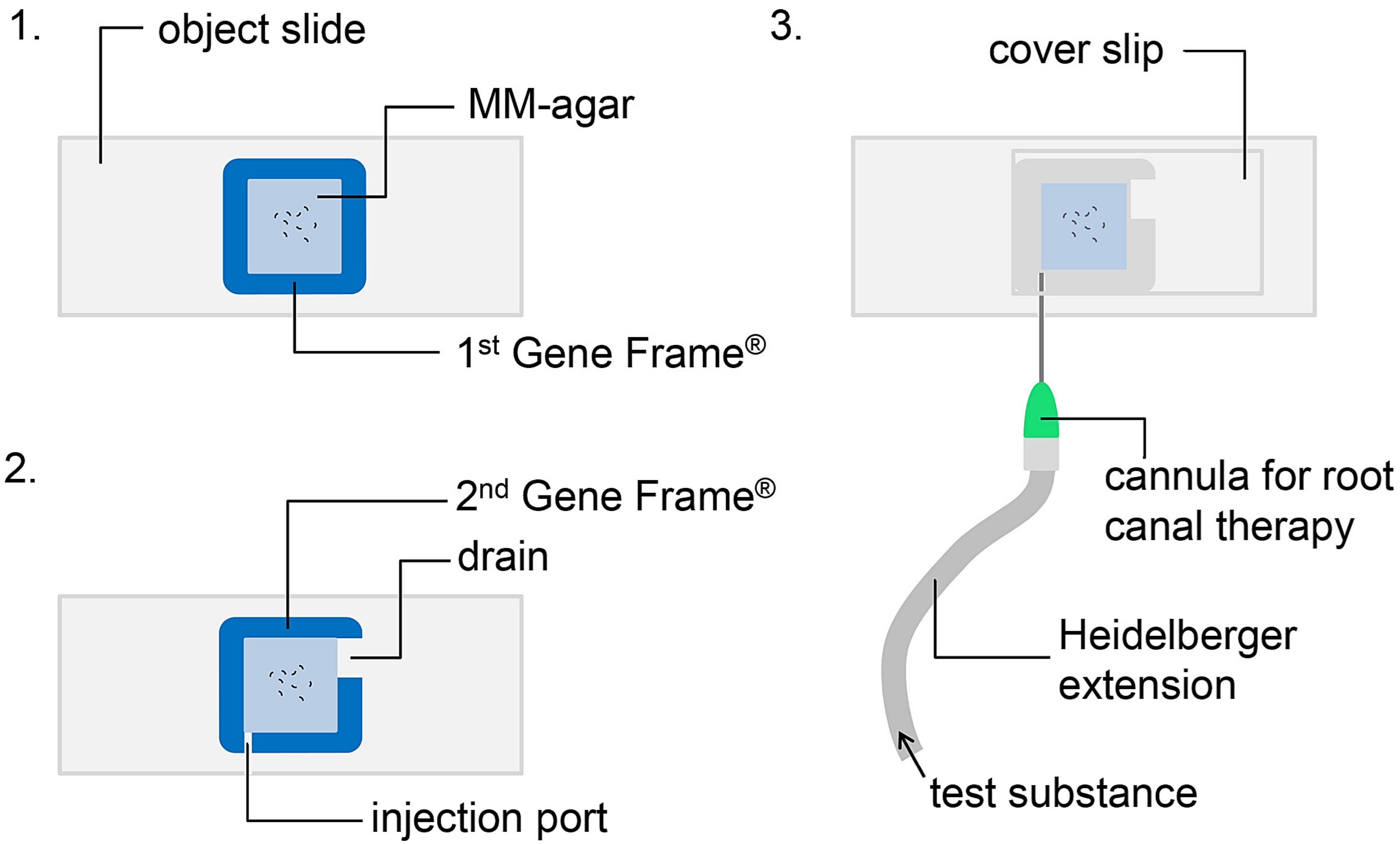


**Figure S5. Flow chamber preparation.** To monitor HyPer-2-responses using confocal laser scanning microscopy a flow chamber was designed that allows simultaneous image acquisition and injection of assay substances, e.g. $H_2O_2$.

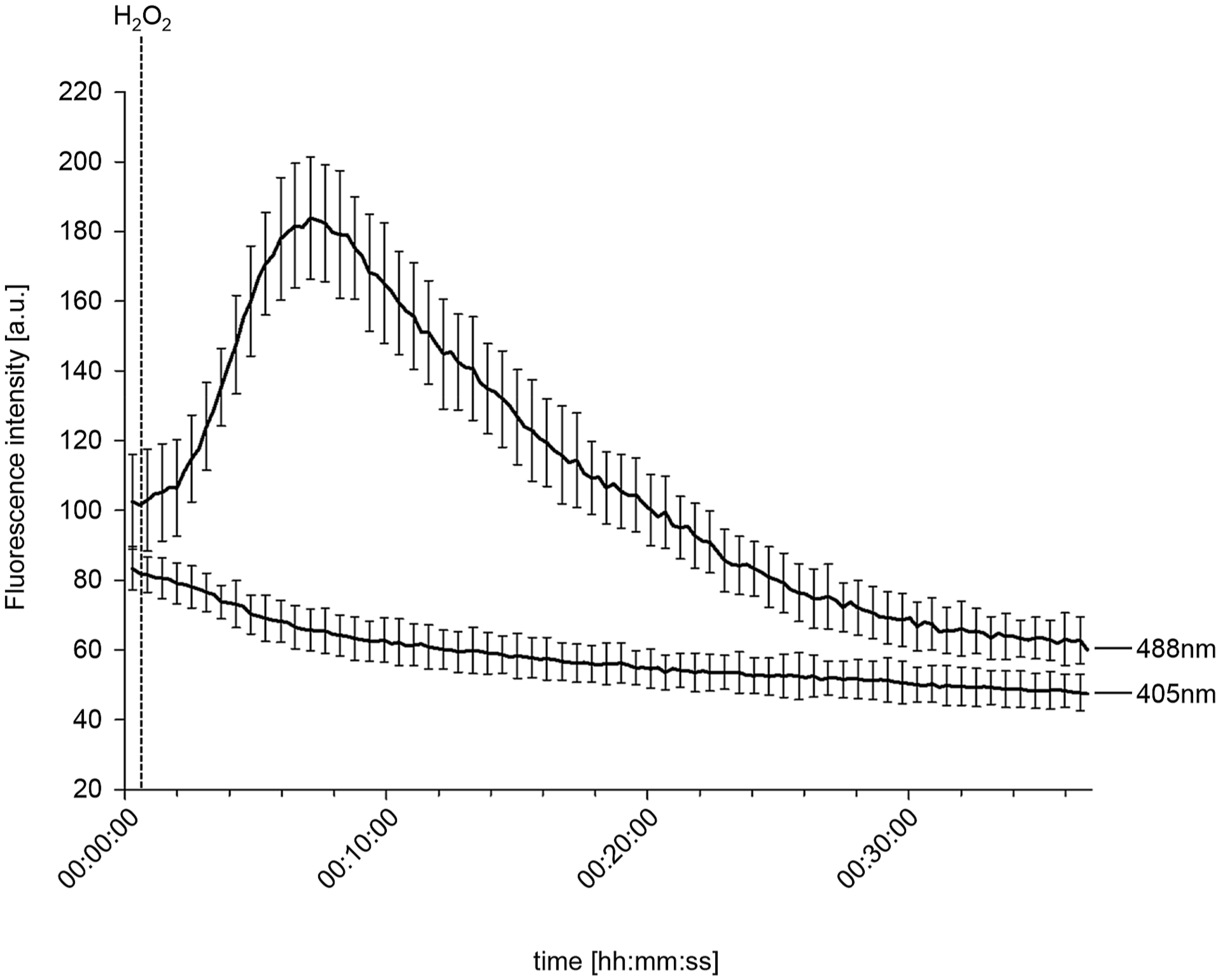


**Figure S6. Fluorescence time course assay of the fungal response to external $H_2O_2$.** Timing of $H_2O_2$ (50 mM) induced changes in HyPer-2-fluorescence intensity after excitation at 405 nm and 488 nm, respectively. Mycelia were raised in a flow chamber (see figure S3) and imaged using confocal laser scanning microscopy. Pixel intensities were measured in three regions of interest marked in a confocal laser scanning time series (see video S1). Error bars represent the standard deviation (n=3).

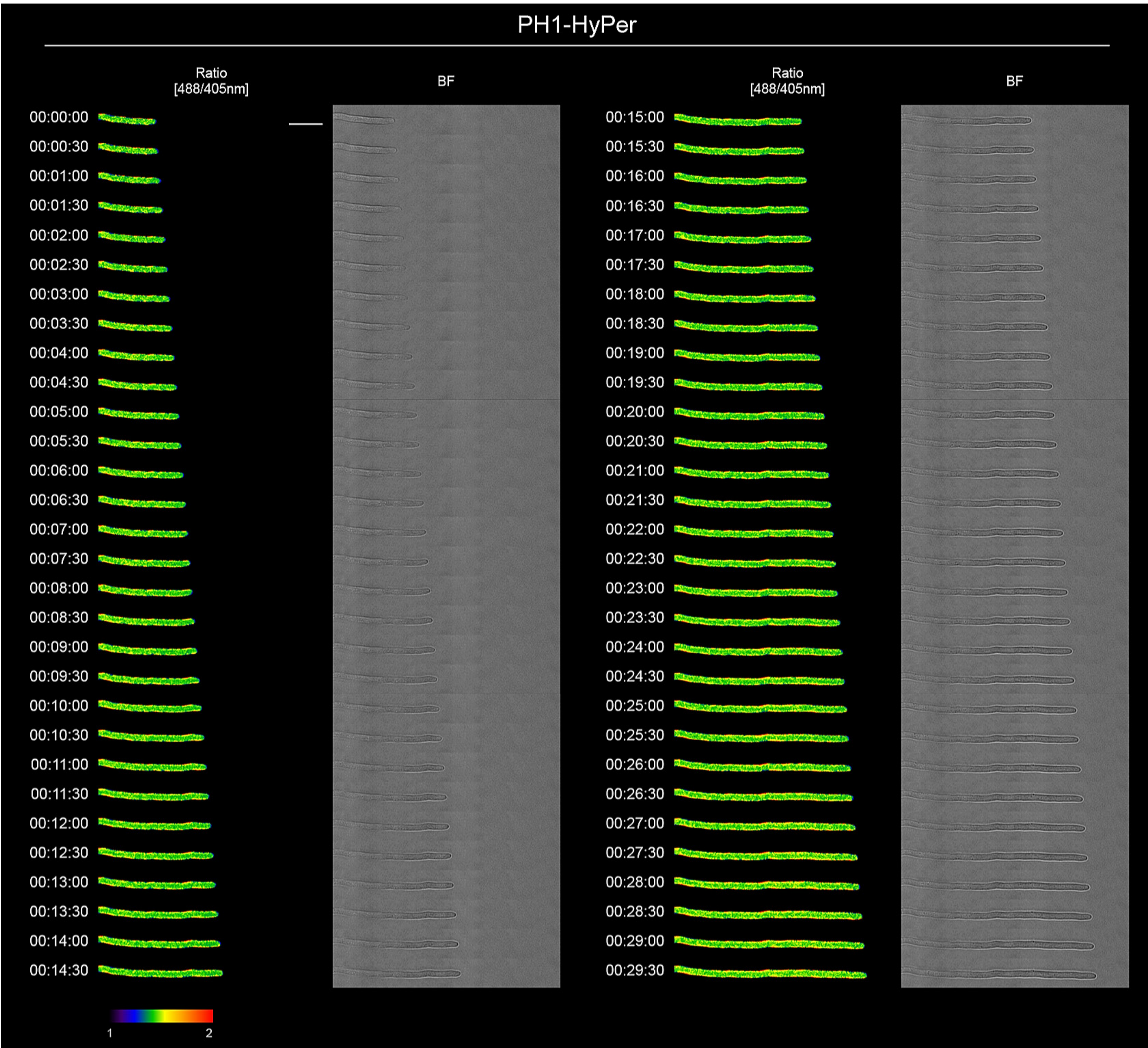


**Figure S7. Ratiometric time course assay of fungal tip growth.** Timing of $H_2O_2$ fluctuations (represented by the ratio [488/405 nm]) during tip growth of hypha expressing HyPer-2. See also video S2. Scale bar: 10 µm.

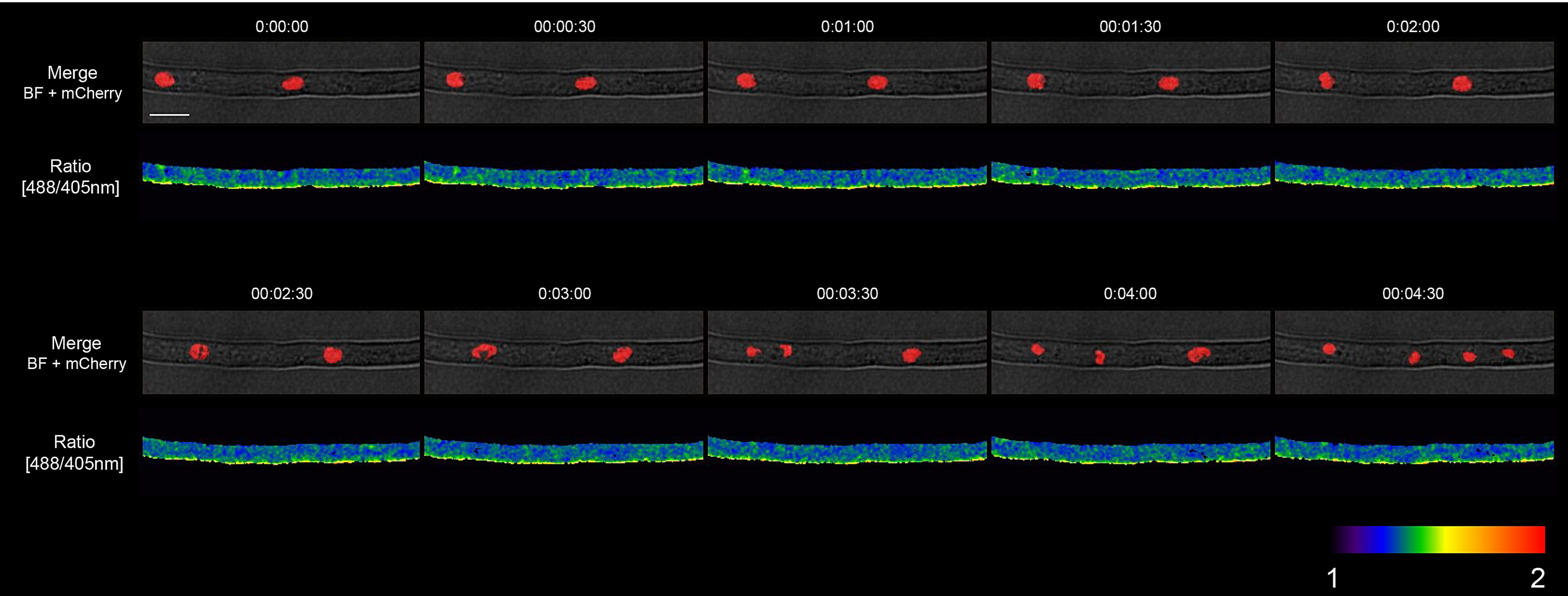


**Figure S8. Ratiometric time course assay of nuclear divisions.** Timing of $H_2O_2$ fluctuations (represented by the ratio [488/405 nm]) during nuclear divisions in hypha expressing HyPer-2. See also video S3. Scale bar: 5 µm.

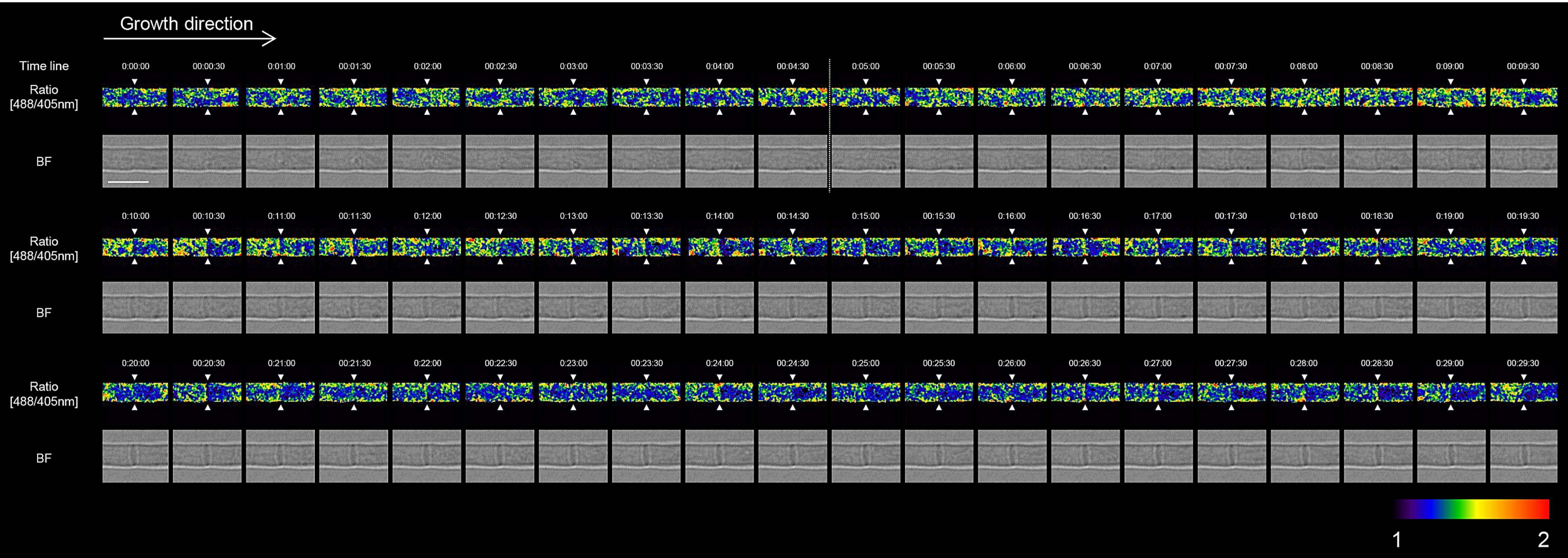


**Figure S9. Ratiometric time course assay of septum formation.** Timing of $H_2O_2$ fluctuations (represented by the ratio [488/405 nm]) during septum formation in hypha expressing HyPer-2. See also video S4. The dotted vertical line represents the initiation of septum formation. Scale bar: 5 µm.

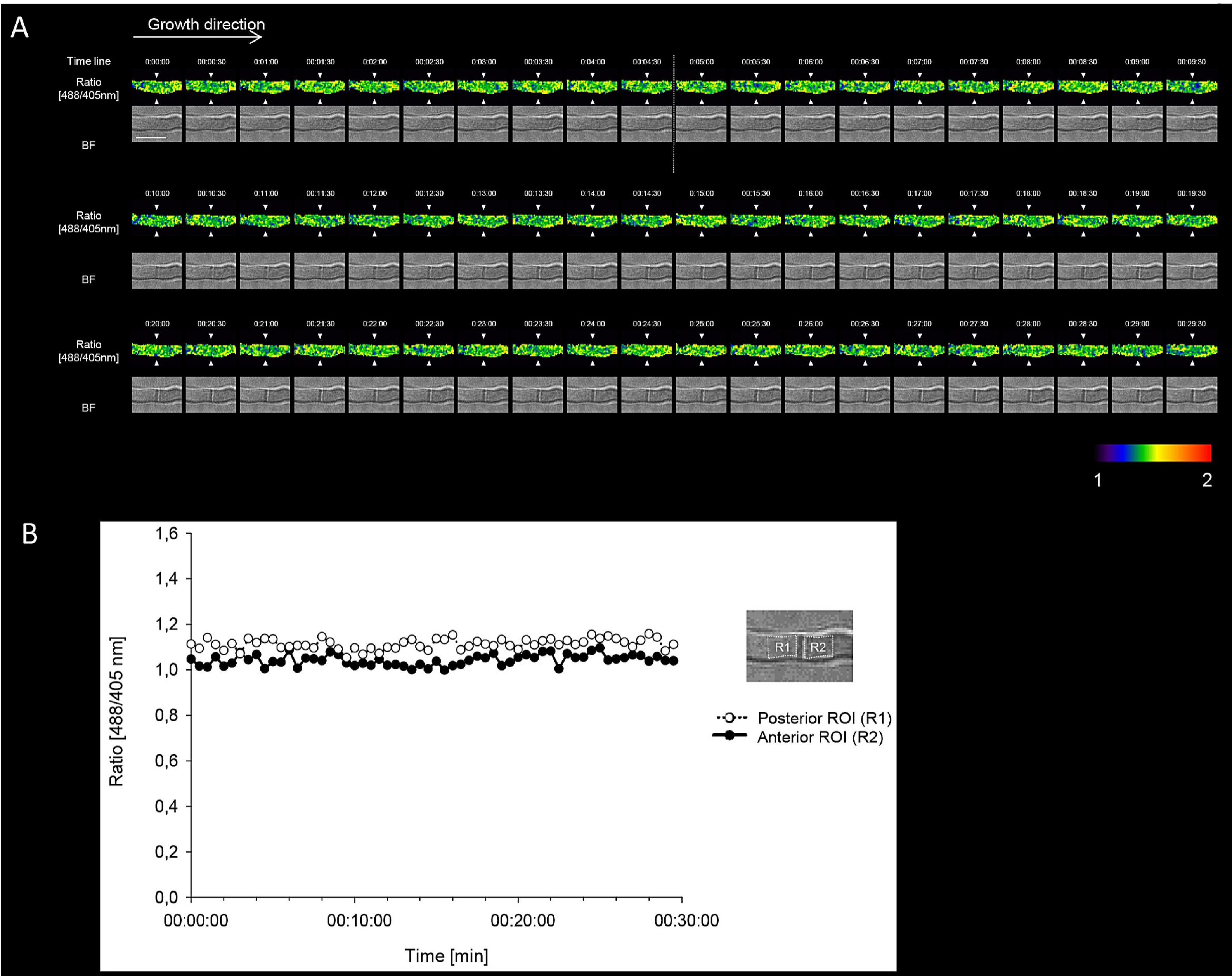


**Figure S10. Ratiometric time course assay of septum formation. A.** Timing of $H_2O_2$ fluctuations (represented by the ratio [488/405 nm]) during septum formation in hypha expressing SypHer. The dotted vertical line represents the initiation of septum formation. Scale bar: 5 µm. **B.** Timing of $H_2O_2$ fluctuations during septum formation. Ratio [488/405 nm] calculated from pixel intensities measurements over time in two regions of interest (ROI R1, covering the posterior part and ROI R2, covering the anterior part of the hypha) marked in a confocal laser scanning time series.

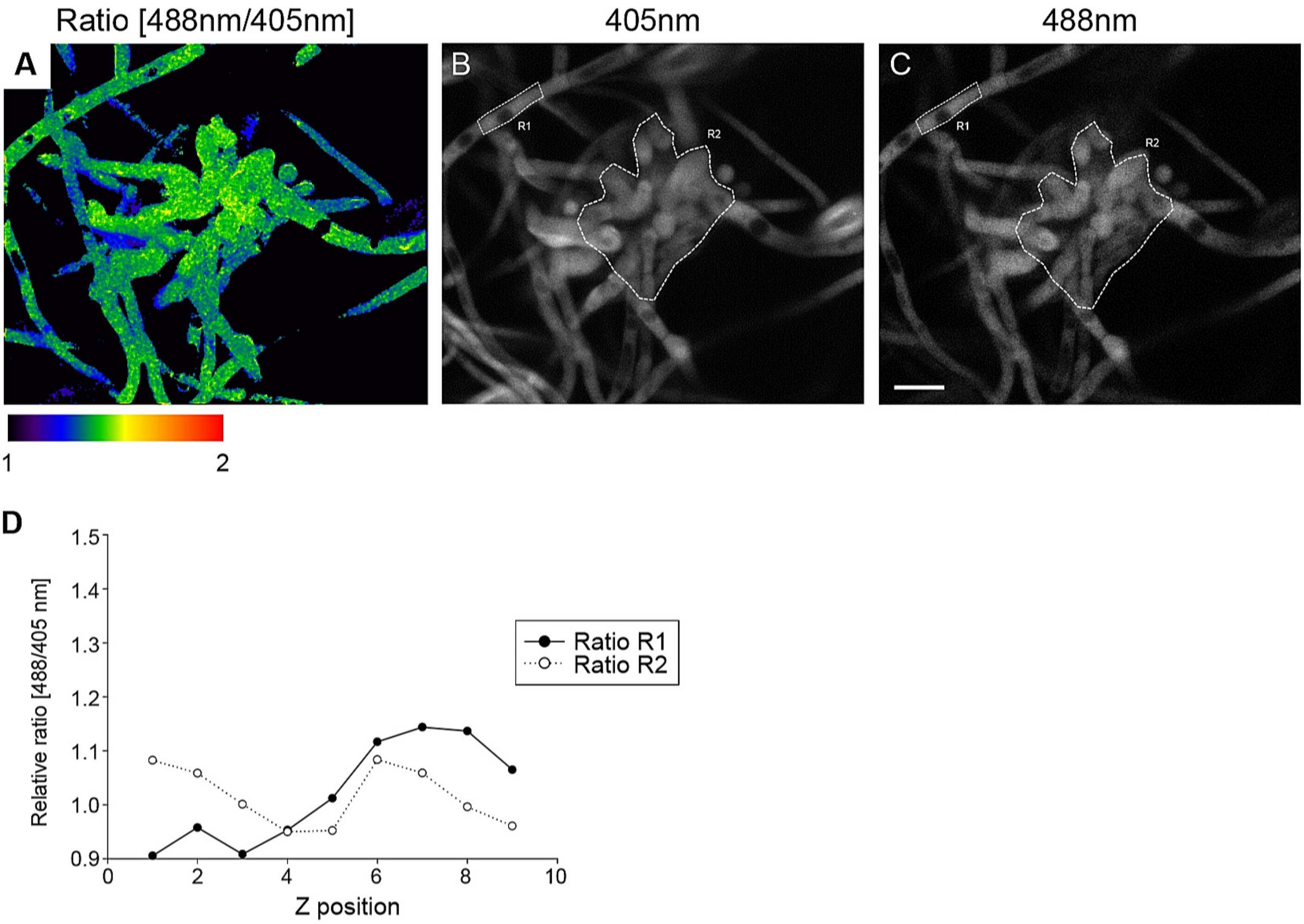


**Figure S11. Ratiometric assay of infection cushion formation in hypha expressing SypHer. A-C.** Average intensity projections of a tiny infection cushion developed on a wheat palea 6 days post inoculation. **A.** Ratio [488/405 nm]. **B.** Fluorescence excited at 405 nm. **C.** Fluorescence excited at 488 nm. **D.** Pixel intensities were measured over nine z-positions in two regions of interest (ROI R1, covering a part of a runner hypha and ROI R2, covering a part of an infection cushion) marked in the confocal laser scanning z-series shown in A and B. Analysis of ten different infection structures gave similar results. Scale bar 10 µm.

| **Primer used for amplification of HyPer and SypHer (*Bam*HI enzyme recognition sites introduced to the primers are underlined)** | | |
|---|---|---|
| **Sequence (5'→ 3')** | **Description** | **Number** |
| GGATCCGGTACCATGGAGATGGCAAGCCCA GCAGGGCGAGACGATGT | Forward primer HyPer/SypHer | A1 |
| GGATCCGCTTTTAAACCGCCTGTT | Reverse primer HyPer/ SypHer | A2 |

**Video legends**

**Video S1. Fluorescence time course assay of the fungal response to external $H_2O_2$.** Time lapse video of germinating conidiospore. $H_2O_2$ (50 mM) supplementation is marked by a red arrow. HyPer-2-fluorescence was recorded after excitation at 405 nm (middle) and 488 nm (left), respectively, and a ratio was calculated (right). Mycelia were raised in a flow chamber (see figure S5) and imaged using confocal laser scanning microscopy.

**Video S2. Fluorescence time course assay of fungal tip growth.** Time lapse video of hyphal growth. HyPer-2-fluorescence was recorded after excitation at 405 nm and 488 nm, respectively, and a ratio was calculated. Mycelia were raised in a flow chamber (see figure S5) and imaged using confocal laser scanning microscopy.

**Video S3. Fluorescence time course assay of nuclear divisions.** Time lapse video of nuclear divisions. HyPer-2-fluorescence was recorded after excitation at 405 nm and 488 nm, respectively, and a ratio was calculated. Mycelia were raised in a flow chamber (see figure S5) and imaged using confocal laser scanning microscopy.

**Video S4. Fluorescence time course assay of septum formation. Time lapse video of septum formation.** HyPer-2-fluorescence was recorded after excitation with 405 nm and 488 nm, respectively, and a ratio was calculated. Mycelia were raised in a flow chamber (see figure S5) and imaged using confocal laser scanning microscopy.
